# Identification of high-risk patients for development of type B aortic dissection based on novel morphological parameters

**DOI:** 10.3389/fphys.2023.1065805

**Published:** 2023-02-02

**Authors:** Da Li, Jiarong Wang, Jichun Zhao, Tiehao Wang, Xiangguo Zeng, Tinghui Zheng, Ding Yuan

**Affiliations:** ^1^ Department of Applied Mechanics, Sichuan University, Chengdu, China; ^2^ Yibin Institute of Industrial Technology, Sichuan University Yibin Park, Yibin, China; ^3^ Division of Vascular Surgery, Department of General Surgery, West China Hospital, Sichuan University, Chengdu, China; ^4^ Med-X center for informatics, Sichuan University, Chengdu, China

**Keywords:** aortic dissection, morphology, predictors, statistics, aortic arch

## Abstract

**Background:** Predicting the development of sporadic type B aortic dissection (TBAD) always remains a difficult issue. This study aimed to identify high-risk patients for development of TBAD based on morphological parameters.

**Methods:** This propensity-score-matched case-control study collected and reconstructed the computed tomography angiography of acute TBAD patients and hospital-based control participants without aortic dissection from January 2013 to December 2016. Multivariate regression analysis was used to calculate the adjusted odds ratio (aOR) and 95% confidence interval (CI). Discriminant and reclassification abilities were compared between our model and a previously established model.

**Results:** Our study included 76 acute TBAD patients and 79 control patients (48 cases and 48 controls after propensity-score matching). The degree of question mark (aOR 1.07, 95% CI 1.04–1.11), brachiocephalic trunk diameter (aOR 1.49, 95% CI 1.20–1.85), brachiocephalic trunk angle (aOR 0.97, 95% CI 0.94–0.99), aortic root diameter (aOR 1.31, 95% CI 1.15–1.48), and aortic width (aOR 1.12, 95% CI 1.07–1.17) were associated with a significantly increased risk of TBAD formation. Similar findings were observed in the propensity-score matching and sensitivity analysis only including hyperacute TBAD patients. A novel prediction model was established based on the aforementioned parameters. The new model showed significantly improved discriminant ability compared with the previously established model (c-index 0.78 [95% CI 0.71–0.85] vs. 0.67 [95% CI 0.58–0.75], *p* = .03), driven by increased reclassification ability in identifying TBAD patients (NRI for events 0.16, 95% CI 0.02–0.30, *p* = .02).

**Conclusion:** Morphological predictors, including the degree of question mark, aortic width, aortic root diameter, brachiocephalic trunk angle, and brachiocephalic trunk diameter, may be used to identify patients at high risk of TBAD.

## Introduction

Acute aortic dissection represents one of the most common types of aortic emergency, defined as the separation of the intimal and medial layers caused by intramural bleeding (Erbel et al., 2014), allowing blood to flow between parietal layers. Typically, the channels between the two layers are referred to as tears, and the new lumen is called the false lumen. According to data from the International Registry of Acute Aortic Dissection (IRAD) registry (Pape et al., 2015), incidences of type B aortic dissection (TBAD) account for around one-third of all dissection patients over a 17-year period. A recent large prospective cohort study reported that the incidence of TBAD may increase, reaching approximately 15 per 100,000 patient years (Landenhed et al., 2015).

The etiology of TBAD is considered to be multifactorial, involving endogenous factors, e.g., Marfan syndrome and Loeys–Dietz syndrome, and exogenous risk factors like hypertension and illicit drug usage (Lombardi et al., 2020). However, these well-acknowledged risk factors seem to be broad and only cover part of TBAD patients, and identification of additional characteristics that could help in predicting the development of TBAD is of great importance. Recently, emerging evidence demonstrated the role of anatomic parameters in association with the risk of TBAD (Shirali et al., 2013; Craiem et al., 2017; Cao et al., 2020; Qiu et al., 2020). A number of studies suggested that larger width and height of the aortic arch or ascending aorta were associated with an increased risk of TBAD (Cao et al., 2020; Qiu et al., 2020), but concern was also raised regarding the usage of post-dissection computed tomography angiography (CTA), in that the morphology of aorta can change significantly after 14 days onset of dissection (Rylski et al., 2014; Peterss et al., 2016). Other studies found that aortic arc angulation or tortuosity might act as potential specific predictors of TBAD in addition to aortic dilation and elongation (Shirali et al., 2013; Cao et al., 2020). However, there was also concern that aortic morphology is age-dependent and should be properly adjusted (Craiem et al., 2017). While previous studies have significantly contributed to our understanding of anatomical parameters associated with development of TBAD, it is still meaningful to explore potential morphologic risk factors that can better predispose individuals at high risk of TBAD in a population of acute TBAD.

A recent study proposed a novel anatomic parameter, i.e., degree of question mark, depicting the composite morphology of the aortic arc and descending aorta (Li et al., 2020). In the present study, we analyzed the reconstructed CTA data from acute TBAD patients and propensity score-matched controls to minimize the confounding effect of age and morphological change of the aorta after dissection. Our aim was 1) to investigate the association between degree of question mark and other anatomic parameters and the risk of development of TBAD, and 2) to develop and validate a new predicting model regarding the development of TBAD.

## Materials and methods

### Study design and settings

In this retrospective matched case-control study, we reviewed the tertiary institutional database for patients who were diagnosed with acute TBAD from January 2013 to December 2016. The local Institutional Review Board approved this study and waived the need for patient consent due to the retrospective review of the anonymous data. Our study was conducted in accordance with the Strengthening the Reporting of Observational Studies in Epidemiology (STROBE) statement of case-control studies (Supplementary Table S1) (von Elm et al., 2007).

### Study population

Case patients were identified as patients who were diagnosed with acute TBAD in tertiary centers. The chronicity classification of TBAD was based on the reporting standards of the Society for Vascular Surgery/Society of Thoracic Surgeons (SVS/STS) (Lombardi et al., 2020). The acute phase of TBAD was defined as the onset of symptoms within 14 days, while hyperacute TBAD was characterized by onset of symptoms within 24 h. Patients were excluded if they had a history of previous aortic diseases, connective tissue diseases, or known genetically triggered aortic diseases (Marfan syndrome, Loeys–Dietz syndrome, and Ehlers–Danlos syndrome); family history of aortic diseases; or were without original CTA records.

Control patients were those who had normal aorta confirmed by aortic CTA, matched by an index date corresponding to the date of TBAD diagnosis. Those patients received CTA for various reasons, for instance, chest pain, splanchnic artery aneurysm or dissection, infrarenal abdominal aortic aneurysm, and peripheral artery diseases.

### Imaging analysis

DICOM (Digital Imaging and Communications in Medicine standard) data of CTA records in case and control groups were extracted for further analysis in an anonymous manner. One author blinded to both patient clinical characteristics and statistical analyses reconstructed DICOM data using image processing software (Mimics v15.0, Materialize, Leuven, Belgium). A centerline was created from the aortic valve annulus to the bifurcation of the abdominal aorta. The reconstructed aorta was divided into nine zones based on the SVS/STS aortic dissection classification system (Lombardi et al., 2020).

### Morphological parameters

**FIGURE 1:**
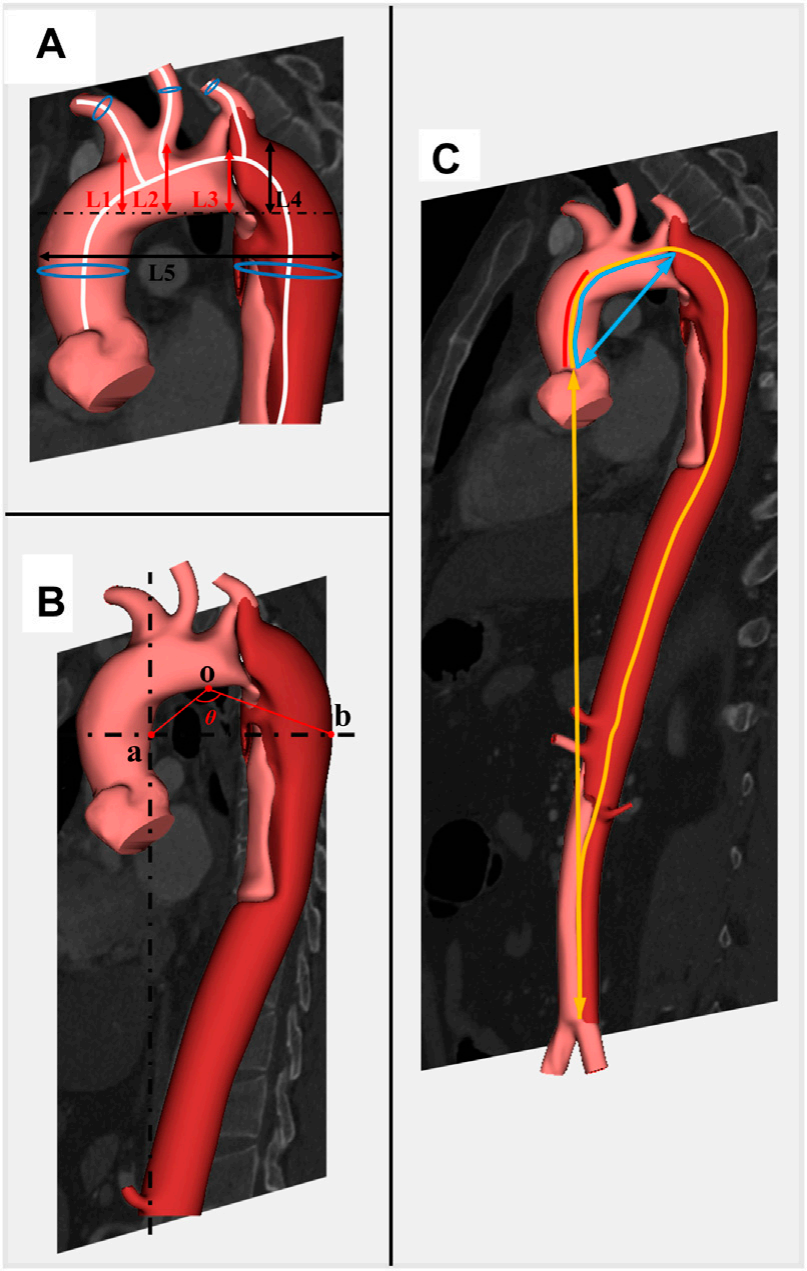
Detailed illustration of morphological parameters related to development of TBAD. Caption: BCT, brachiocephalic trunk; LCA, left carotid artery; LSA, left subclavian artery. **(A)** L1, L2, and L3 represent the height of BCT, LCA, and LSA, respectively; L4 and L5 represent the height of aortic arch and the width of aorta, respectively; blue circles represent the diameters on the centerline. **(B)** Angle θ represents the degree of question mark. **(C)** Centerlines of red, blue, and yellow represent the length of the ascending aorta, ascending aorta and aortic arch, and total aorta; the blue straight line represents the distance between the aortic root and the aortic arch; the yellow straight line represents the distance between the aortic root and the end of descending aorta.

The diameter for the brachiocephalic trunk (BCT), left carotid artery (LCA), and left subclavian artery (LSA) was measured 5 mm away from the aortic arch. The length of the aorta was measured from the centerline from the aortic valve annulus to the bifurcation of the abdominal aorta. The height for BCT, LCA, and LSA was measured by the vertical distance from the position of these branching arteries to the level of the highest aortic arch. The angle of BCT, LCA, and LSA was measured by the angle between the centerline of the branch artery and the centerline of the aorta. Aorta width was defined by the maximum distance between the ascending and descending aorta. Aorta height was defined as the vertical distance from the highest point of the outer curvature of the aortic arch to the highest point of the inner curvature of the aortic arch. As described in a recent study (Li et al., 2020), the question mark was defined as follows: first, find the highest point of the aortic arch defined as o, then find the most distal point in the horizontal direction of the descending aorta defined as b and the horizontal plane in which point B is located, then find the projection a of the entrance of the celiac artery in this horizontal plane, and finally the angle formed by oa and ob is the question mark. Aortic tortuosity was defined as the ratio of the straight-line distance of the aorta to the centerline length of the aorta. A detailed illustration of morphological measurement is displayed in Figure 1.

### Statistical analyses

Continuous data were presented as mean ± standard deviation (SD) if they were normally distributed or as median (interquartile range [IQR]) and *vice versa*. Categorical data were presented as numbers (percentages). Student’s *t-*test or Mann–Whitney *U* test was used for univariate analysis of continuous data, and the χ2 test or Fisher’s exact test was used for categorical data. Multiple imputation was adopted to handle missing data of confounders. R studio Version 1.2.1335 (http://www.R-project.org) and Empower (www.empowerstats.com, X&Y Solutions, Inc., Boston, MA) were utilized for statistical analysis.

### Propensity-score matching

To reduce the potential bias generated by confounding factors, we performed multivariate logistic regression adjusting for age, gender, BMI, and anatomical characteristics in the overall population. Furthermore, we performed the sensitivity analysis in three fashions. First, we conducted a propensity-score (PS) matching analysis, based on age, gender, BMI, hypertension, and other comorbidities, to further account for the confounding effect. Second, we re-ran our analysis by only including hyperacute TBAD patients and 1:1 PS-matched control patients, as the morphology of the hyperacute dissected aorta is considered to be similar to that of the pre-dissected aorta (Peterss et al., 2016; Lombardi et al., 2020). Third, we excluded degenerative infrarenal abdominal aortic aneurysms in the overall controls in order to remove the potential confounding effect of abdominal aortic wall degeneration on the whole aortic morphology.

### Model development and validation

The receiver operating characteristic (ROC) curve was drawn to depict the predicting ability of different predictors on the development of TBAD. Independent predictors identified by multivariate logistic regression analysis were involved in the full model, and the final model was fitted using stepwise backward selection (Collins et al., 2015). The discriminant ability of the model was assessed using Harrell’s concordance statistic (c-index). For an individual patient, the risk of development of TBAD was calculated using the following formula:

$$P\left(risk\;of\;TBAD\right)=\frac{\exp\left(LP\right)}{1+\exp\left(LP\right)}\;,$$

where LP (linear predictor) is the sum of the products of the predictors and associated coefficients for a certain patient.

The internal validation was performed using bootstrapping with 1,000 resampling methods. Agreement between predicted and observed events was assessed graphically by calibration curves, which presented a visual estimation of the model’s performance. If the points fall on or near the 45° line, the model is considered to have good calibration. If the points fall above the 45° line, the model is considered to overestimate the risk of TBAD and *vice versa*.

### Clinical implication

To evaluate the implication of our model in clinical practice, we compared the discriminant ability and reclassification ability of our model (Model 2) with a previously established model (Model 1) (Shirali et al., 2013). Harrell’s concordance statistic (c-index) was used to assess the discriminatory ability, and the net reclassification index (NRI) was applied to evaluate the reclassification performance of each model (Pencina et al., 2008). Decision curve analysis was used to assess the clinical benefit of Model 2 versus Model 1, which was presented as the net benefit (Vickers et al., 2016). A value of 0 represents no benefit, and higher values indicate greater benefit.

## Results

### Baseline characteristics

The study population consisted of 76 acute TBAD patients and 79 control patients. A detailed selection flow diagram is shown in Supplementary Figure S1. The case group participants were found to be significantly older than the control group participants (65.03 ± 12.30 vs. 51.17 ± 12.11, *p* < .001). No significant difference was found between the two groups regarding BMI, gender, hypertension, and other major comorbidities (Table 1). After propensity-score matching, 48 case patients and 48 control patients were matched in the further analyses, with a good balance in age, gender, BMI, and comorbidities (Table 1). Among the acute TBAD patients, the median onset time was 19 h, and 30 patients were found to be hyperacute.

**TABLE 1:** Baseline characteristics of the included population.

	Overall population			PS-matched population		
	Acute TBAD (N = 76)	Control (N = 79)	*p*-value	Acute TBAD (N = 48)	Control (N = 48)	*p*-value
Baseline characteristics						
Age	51.17 ± 12.11	65.03 ± 12.30	<.001	57.23 ± 10.22	58.96 ± 10.75	.42
BMI	25.72 ± 5.77	25.76 ± 6.03	.97	25.19 ± 5.70	25.92 ± 6.26	.55
Gender			.18			.81
Female	18 (23.68%)	12 (15.19%)		12 (25.00%)	11 (22.92%)	
Male	58 (76.32%)	67 (84.81%)		36 (75.00%)	37 (77.08%)	
Hypertension	45 (59.21%)	48 (60.76%)	.84	29 (60.42%)	25 (52.08%)	.41
COPD	20 (26.32%)	29 (36.71%)	.16	15 (31.25%)	18 (37.50%)	.52
CKD	13 (17.11%)	10 (12.66%)	.43	8 (16.67%)	4 (8.33%)	.22
Morphological parameters						
Question mark	56.32 ± 18.60	43.76 ± 11.61	<.001	54.59 ± 15.27	44.32 ± 12.56	<.001
BCT diameter	13.79 ± 2.08	12.70 ± 1.89	<.001	13.93 ± 2.20	12.58 ± 1.95	.002
LCA diameter	9.26 ± 2.00	8.68 ± 1.30	.033	9.45 ± 2.19	8.66 ± 1.41	.037
LSA diameter	10.95 ± 1.73	10.88 ± 1.66	.78	11.02 ± 1.70	10.63 ± 1.53	.24
Ascending aortic length	62.94 ± 11.18	64.70 ± 9.43	.29	65.72 ± 10.80	63.52 ± 10.68	.32
BCT height	40.74 ± 9.62	43.72 ± 8.79	.046	42.54 ± 9.86	41.62 ± 9.04	.63
LCA height	27.94 ± 9.57	30.96 ± 8.83	.043	30.36 ± 10.26	29.19 ± 9.35	.56
LSA height	14.24 ± 7.72	16.11 ± 7.89	.14	16.44 ± 7.78	15.25 ± 6.98	.43
Aortic width	116.03 ± 14.59	110.21 ± 13.37	.011	120.77 ± 13.55	107.89 ± 13.18	<.001
Aortic height	26.78 ± 24.89	25.47 ± 9.51	.35	27.21 ± 30.69	26.01 ± 8.47	.11
Centerline length of the aorta	514.31 ± 39.81	517.31 ± 32.90	.61	520.72 ± 40.02	506.51 ± 31.85	.06
Linear length of aorta	272.86 ± 22.28	261.89 ± 28.23	.008	266.75 ± 20.23	261.85 ± 22.13	.26
BCT angle	58.16 ± 15.13	62.79 ± 13.54	.046	55.70 ± 13.93	63.11 ± 13.24	.009
LSA angle	70.63 ± 16.33	69.41 ± 14.62	.62	66.90 ± 15.97	70.76 ± 14.92	.22
Aortic root diameter	32.51 ± 4.50	30.34 ± 3.85	.002	33.80 ± 4.23	29.92 ± 3.81	<.001
Aortic arch diameter	29.23 ± 4.06	28.20 ± 3.42	.09	30.20 ± 3.10	27.95 ± 3.79	.002
Aortic tortuosity	1.90 ± 0.19	2.02 ± 0.48	.041	1.96 ± 0.19	1.95 ± 0.18	.69

Continuous variables are presented as mean ± SD, and categorical variables are presented as n (%). PS, propensity score; TBAD, type B aortic dissection; BMI, body mass index; CKD, chronic kidney disease; COPD, chronic obstructive pulmonary disease; BCT, brachiocephalic trunk; LCA, left carotid artery; LSA, left subclavian artery.

### Association between morphological parameters and TBAD

In the overall population, the degree of question mark, BCT diameter, LCA diameter, BCT height, LCA height, aortic width, aortic root diameter, BCT angle, and aortic tortuosity were associated with risk of TBAD in the univariate analyses (Table 2). After multivariate adjustment, the degree of question mark (OR 1.06, 95% CI 1.03–1.09, *p* < .001), BCT diameter (OR 1.32, 95% CI 1.11–1.56, *p* = .001), aortic width (OR 1.14, 95% CI 1.08–1.21, *p* < .001), BCT angle (OR 0.97, 95% CI 0.94–0.99, *p* = .020), and aortic root diameter (OR 1.31, 95% CI 1.15–1.48, *p* < .001) remained the independent predictors of the development of TBAD (Table 2).

**TABLE 2:** Results of univariate and multivariate logistic regression analysis regarding the risk of TBAD in the overall population.

	Univariate analysis	*p*-value	Multivariate analysis	*p*-value
Gender	0.58 (0.26, 1.30)	.18		
Age	0.92 (0.89, 0.95)	<.001		
BMI	1.00 (0.95, 1.05)	.97		
Question mark	1.06 (1.03, 1.09)	<.001	1.07 (1.04, 1.11)	<.001
BCT diameter	1.32 (1.11, 1.56)	.001	1.49 (1.20, 1.85)	<.001
LCA diameter	1.24 (1.01, 1.52)	.038	1.20 (0.93, 1.54)	.15
LSA diameter	1.03 (0.85, 1.24)	.78		
Ascending aortic length	0.98 (0.95, 1.01)	.29		
BCT height	0.97 (0.93, 1.00)	.048	1.00 (0.96, 1.05)	.91
LCA height	0.96 (0.93, 1.00)	.045	1.02 (0.97, 1.06)	.51
LSA height	0.97 (0.94, 1.00)	.05		
Aortic width	1.03 (1.01, 1.05)	.012	1.12 (1.07, 1.17)	<.001
Aortic height	1.00 (0.99, 1.02)	.67		
Centerline length of the aorta	1.00 (0.99, 1.01)	.61		
Linear length of the aorta	1.02 (1.00, 1.03)	.011	1.01 (0.99, 1.02)	.42
BCT angle	0.98 (0.96, 1.00)	.049	0.97 (0.94, 0.99)	.020
LSA angle	1.01 (0.98, 1.03)	.62		
Aortic root diameter	1.14 (1.05, 1.23)	.003	1.31 (1.15, 1.48)	<.001
Aortic arch diameter	1.08 (0.99, 1.18)	.09		
Aortic tortuosity	0.12 (0.02, 0.72)	.020	0.41 (0.04, 3.89)	.43

Note: PS, propensity score; TBAD, type B aortic dissection; BMI, body mass index; CKD, chronic kidney disease; COPD, chronic obstructive pulmonary disease; BCT, brachiocephalic trunk; LCA, left carotid artery; LSA, left subclavian artery. Variates adjusted in multivariate logistic regression analysis: age, gender, BMI, aortic height, and centerline length of the aorta.

After PS matching, we found that the degree of question mark (OR 1.06, 95% CI 1.03–1.10, *p* < .001), BCT diameter (OR 1.41, 95% CI 1.12–1.78, *p* = .003), aortic width (OR 1.11, 95% CI 1.06–1.17, *p* < .001), aortic root diameter (OR 1.34, 95% CI 1.14–1.58, *p* < .001), and aortic arch diameter (OR 1.22, 95% CI 1.06–1.41, *p* = .006) were associated with a significantly increased risk of TBAD, while BCT angle was associated with a significantly decreased risk of TBAD (OR 0.95, 95% CI 0.91–0.98, *p* = .002). In the subgroup of hyperacute TBAD and sensitivity analysis of non-degenerative controls, similar results were observed (Supplementary Table S3).

### Model development

All morphological parameters with a significant (*p* < .05) univariate association with the risk of TBAD were fitted into a multivariate model, after which stepwise backward selection was performed. The degree of question mark, BCT angle, BCT diameter, aortic root diameter, and aortic width were included in the final model, and the following formula summarized the probability of development of TBAD:

$$P\left(risk\;of\;TBAD\right)=\frac{\exp\left(LP\right)}{1+\exp\left(LP\right)}\;,$$

where LP = −5.97378 + 0.05570* degree of question mark + 0.05196* aortic root diameter + 0.00772* aortic width + 0.16872* BCT diameter −0.02608* BCT angle.

### Model validation

**FIGURE 2:**
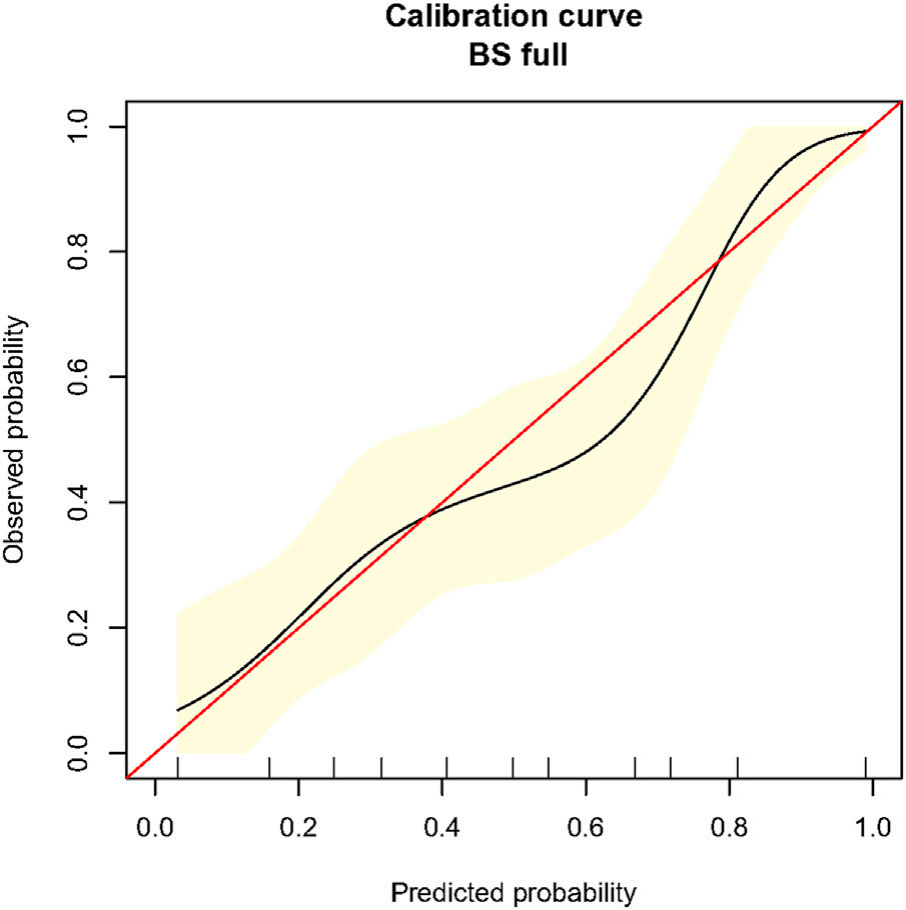
Calibration curve for the novel prediction model in propensity-score-matched population. Caption: BS, bootstrapping. The red line is the ideal reference calibration line, and the black curve is the actual observed calibration curve. The x-axis indicates the predicted risk of TBAD formation, and the y-axis indicates the observed risk of TBAD formation. The yellow area indicates the 95% confidence interval of the observed risk generated from 1,000 time bootstrapping.

The C-index of the predictive model was 0.78 (95% CI 0.71–0.85). Figure 2 presents a graphical representation of the calibration curve from internal validation with 1,000 bootstrapping. The curve shows overall agreement between the predicted and observed risk of development of TBAD.

### Clinical utility

**FIGURE 3:**
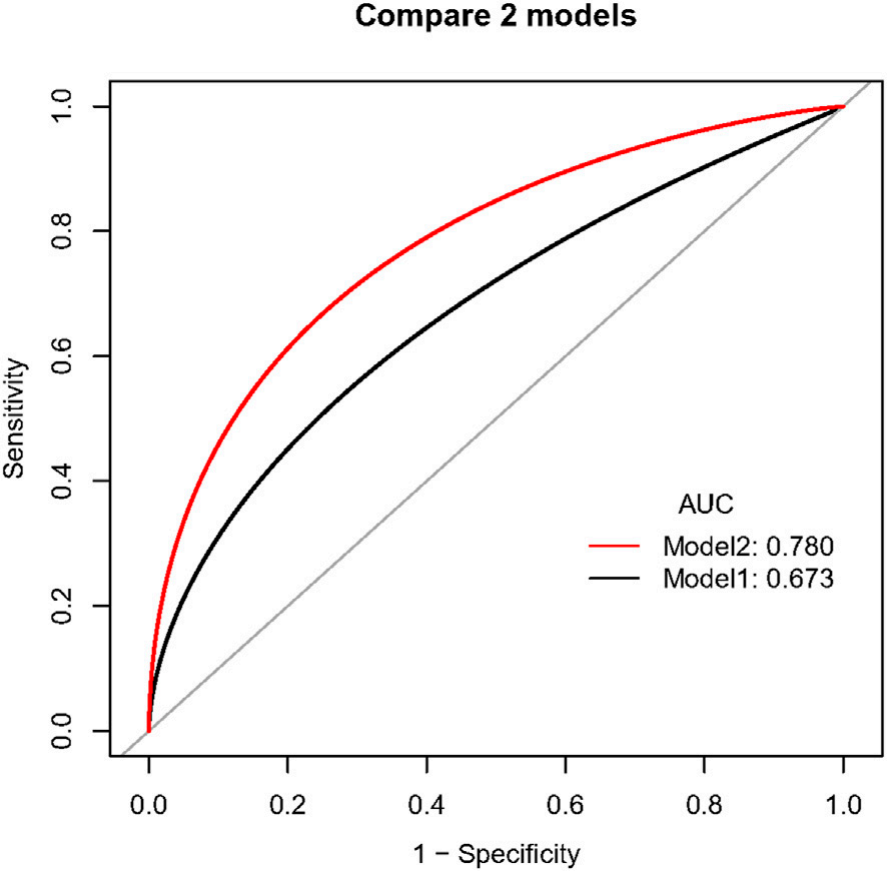
Comparison of discriminatory ability between two prediction models in the overall population. Caption: Model 1 included BCT angle, aortic tortuosity, and aortic arch diameter. Model 2 involved the degree of question mark, aortic root diameter, aortic width, BCT angle, and BCT diameter.

**FIGURE 4:**
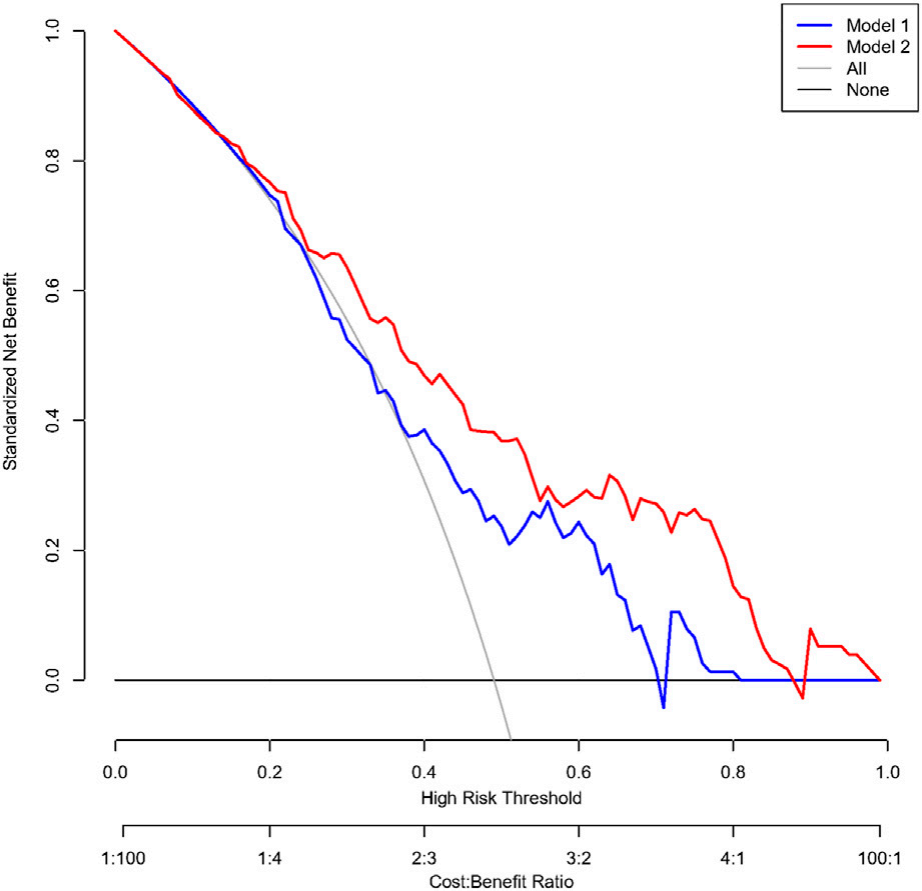
Decision curve analysis comparing the clinical utility between the two models in a propensity-score-matched population. Caption: Model 1 included BCT angle, aortic tortuosity, and aortic arch diameter. Model 2 involved the degree of question mark, aortic root diameter, aortic width, BCT angle, and BCT diameter.

When compared with the previously reported model, our model had significantly improved discriminatory ability (c-index 0.78 [95% CI 0.71–0.85] vs. 0.67 [95% CI 0.58–0.75], *p* = .030, Figure 3) and reclassification ability for TBAD patients (NRI for events 0.16, 95% CI 0.02–0.30, *p* = .015, Supplementary Table S2) compared to the previously established model in the overall population. After PS-matching, we also found significantly improved discriminatory ability in our model (c-index 0.84 [95% CI 0.76–0.90] vs. 0.72 [95% CI 0.62–0.81], *p* = .015, Supplementary Figure S2), driven by increased reclassification ability in identifying non-TBAD patients (NRI for non-events 0.13, 95% CI 0.03–0.23, *p* = .008, Supplementary Table S2). As for clinical performance, the decision curve analysis showed that Model 2 was associated with a higher net benefit between threshold probabilities of 20%–80% (Figure 4), which suggested the superiority of our model in clinical practice.

## Discussion

In this PS-matched case-control study, we identified several novel morphological parameters associated with a significantly increased risk of development of TBAD, including the degree of question mark, BCT diameter, BCT angle, aortic width, and aortic root diameter. We also developed and internally validated a novel prediction model involving the aforementioned parameters to generate individualized risk estimates for development of TBAD. Predicted and observed risks were concordant both in the overall and PS-matched population. Additionally, the novel model had superior discriminatory ability compared to a previously established model, with higher clinical net benefit.

Prediction of TBAD using morphological parameters received increasing attention in the recent decade as it could act as an important supplementary approach to manage the risk of TBAD, in addition to blood pressure control. Due to the multifactorial etiology of TBAD, investigation of morphological predisposing factors should avoid the confounding effect of other risk factors, such as hypertension and potential genetic factors. Our study addressed this issue by excluding patients with a history of previous aortic diseases, connective tissue diseases, known genetically triggered aortic diseases, or family history of aortic diseases. In addition, we performed PS-matching to balance age, gender, and hypertension to minimize the effect of other factors in predicting TBAD.

Prior studies also identified several morphological predictors of TBAD, among which aortic tortuosity represented the most commonly validated parameter (Shirali et al., 2013; Cao et al., 2020). However, we did not observe a significant association between aortic tortuosity and the risk of TBAD in our study. Interestingly, the results of univariate analysis revealed that aortic tortuosity was associated with a decreased risk of TBAD, which is opposite to previous findings (Shirali et al., 2013). These contradictory results may be attributed to the older age of the control group participants in our study, and as is pointed out in prior studies, aortic morphology is age-dependent, and older patients are prone to have larger and more tortuous aorta (Collins et al., 2014; Craiem et al., 2017; Bai et al., 2020). The negative results in the multivariate analysis and PS-matched population further confirmed this issue after adjusting for age, which suggested aortic tortuosity was an index that can be easily affected by age and should be categorized by age in clinical application. By comparison, our study proposed a novel parameter, i.e., degree of question mark, depicting the spatial tortuosity of the aortic arc and aorta. Independent of age distribution, the degree of question mark had superior discriminatory capacity compared to aortic tortuosity. A previous study also found that the degree of question mark also plays an important role in remodeling of TBAD (Li et al., 2020), although the exact underlying biomechanical mechanism remains to be explored in the future.

In addition to parameters related to aortic tortuosity, morphological parameters of BCT (angle and diameter) were also associated with an increased risk of TBAD. Our results were consistent with those of prior studies (Shirali et al., 2013), which further confirmed the role of the tangent angle of BCT originating from the aortic arch in the formation of TBAD. The risk of TBAD decreased with the BCT angle and increased with the BCT diameter. Though the underlying mechanism remained unknown, it was worth noticing that both decreased BCT angle and increased BCT diameter were associated with an increased volume distribution of blood flow in BCT. The association between BCT blood volume and the risk of TBAD warrants further investigation with computational fluid dynamics analysis to shed light on the potential biomechanical etiology of TBAD. Another novel morphological parameter identified in our study was aortic width, which acted as a supplementary illustration to the spatial geometry of the aorta. Previous studies revealed a subtle association between aortic width and adverse cerebrocardiovascular events among community-dwelling adults (Chuang et al., 2018), probably due to the fact that spatial tortuosity may augment the severity and distribution of flow disturbances (Xie et al., 2013; Li et al., 2019).

In order to be widely applicable in clinical practice, those pre-identified morphological risk factors need to be integrated into a risk stratification algorithm. Our newly developed model showed good discrimination and reclassification (as indicated by the c-index and NRI, respectively) and fine agreement between the observed and predicted risk of TBAD (as illustrated by the calibration plots). Additionally, the performance of our model remained stable in PS-matched sensitivity analysis, which further suggested the robustness of the model. Compared with the previous model (Shirali et al., 2013), our model had a superior discriminatory ability with internal validation of 1,000 time bootstrapping; thus, this novel model may provide the clinician with the necessary individualized data to facilitate surveillance protocol regarding the risk of TBAD.

The current study had several limitations. First, concerns may be raised against the potential alteration of morphology after the development of TBAD. Ideally, a prospective observational study is preferred to identify morphological risk factors of TBAD formation, but the long follow-up and low incidence of disease make it unrealistic in actual practice. To address this issue, we performed sensitivity analysis by exclusively including hyperacute TBAD patients, who were assumed to have similar aortic morphology compared with pre-dissected aorta (Peterss et al., 2016; Orabi et al., 2018). The consistent results between overall and sensitivity analysis further confirmed the rationality of our study. Second, the sample size was relatively small, but we used bootstrapping of 1,000 replicates to derive the estimates of confidence intervals of the effect size. Third, though our study matched hypertension between the case and control group, whether a transient hypertensive crisis existed cannot be sure from existing information; hence, the results may be subjective to some unmeasured confounding factors. Fourth, despite the retrospective nature of our study, current findings were hypothesis-generating, which could still be informative and set the stage for prospective validation.

## Conclusion

In this PS-matched case-control study, several morphological parameters, including degree of question mark, BCT diameter, BCT angle, aortic width, and aortic root diameter, were identified as potential anatomic markers used to predispose individuals at a high risk of TBAD. Based on these parameters, we also developed and internally validated a novel prediction model with high discriminant and reclassification ability to derive individualized risk estimates for development of TBAD. External validation of our model in a prospective cohort study is warranted in the future.

## Data Availability

The original contributions presented in the study are included in the article/Supplementary Material; further inquiries can be directed to the corresponding authors.
